# A polygenic score for height identifies an unmeasured genetic predisposition among pediatric patients with idiopathic short stature

**DOI:** 10.21203/rs.3.rs-4921143/v1

**Published:** 2024-10-14

**Authors:** John P. Shelley, Mingjian Shi, Josh F. Peterson, Sara L. Van Driest, Jill H. Simmons, Jonathan D. Mosley

**Affiliations:** Vanderbilt University Medical Center; Vanderbilt University Medical Center; Vanderbilt University Medical Center; National Institutes of Health (NIH); Vanderbilt University Medical Center; Vanderbilt University Medical Center

**Keywords:** Polygenic scores, Height, Common genetic variation, Short stature, Idiopathic short stature

## Abstract

**Background:**

A subset of children with short stature do not have an identified clinical explanation and are assigned a diagnosis of idiopathic short stature (ISS). We hypothesized that a polygenic score for height (PGS_height_) could identify children with ISS who have an unrecognized heritable predisposition to shorter height.

**Methods:**

We examined 534 pediatric participants in an EHR-linked DNA biobank (BioVU) who had undergone an evaluation for short stature by an endocrinologist. We used a previously validated PGS_height_ and standardized it to a standard deviation (SDS) of 1. PGS_height_ differences between short stature subtypes was estimated using Tukey’s HSD. The PGS_height_ and mid-parental height (MPH) were then used to predict adult heights for each participant and these predictions were compared using Cohen’s *d* stratifying by short stature subtype. The ability of the PGS_height_ to discriminate between ISS and short stature due to underlying disease was evaluated using logistic regression models with area under the ROC curve (AUC) analyses and testing the incremental benefit (ΔAUC) of adding the PGS_height_ to prediction models.

**Results:**

Among the 534 participants, 22.1% had ISS (median [IQR] PGS_height_ SDS = −1.31 [−2.15 to −0.47]), 6.6% had familial (genetic) short stature (FSS) (−1.62 [−2.13 to −0.54]), and 45.1% had short stature due to underlying pathology (−0.74 [−1.23 to −0.19]). Children with ISS had similar PGS_height_ values as those with FSS (ΔPGS_height_ [95% CI] = 0.19 [−0.31 to 0.70], *p* = 0.75), but predicted heights generated by the PGS_height_ were lower than the MPH estimate for children with ISS (*d* = −0.64; *p* = 4.0×10^−18^) but not FSS (*d* = 0.05; *p* = 0.46), suggesting that MPH underestimates height in the ISS group. Children with ISS had lower PGS_height_ values than children with pathology (ΔPGS_height_ = −0.60 SDS [−0.89 to −0.31], p < 0.001), suggesting children with ISS have a larger predisposition to shorter height. In addition, the PGS_height_ improved model discrimination between ISS and pathologic short stature (ΔAUC, + 0.07 [95% CI, 0.01 to 0.11]).

**Conclusions:**

Some children with ISS have a clinically unrecognized polygenic predisposition to shorter height that is comparable to children with FSS and larger than those with underlying pathology. A PGS_height_ could help clinicians identify children who have a benign predisposition to shorter height.

## Introduction

There has been considerable interest in translating polygenic scores for use in clinical settings to guide care decisions.^1^ To date, these applications have largely focused on polygenic risk scores developed for the goal of identifying individuals at risk for adult-onset diseases, such as cancers or heart diseases.^2^ There have been few clinical applications of these scores in pediatric patients, partly because many polygenic influences on clinical outcomes are age-dependent and scores are calibrated to predict these influences in adulthood.^3–6^ Here, we describe an application of a polygenic score for adult height (PGS_height_) in children being evaluated for short stature.

The clinical population of children with short stature is heterogeneous, as short stature can be caused by a benign genetic predisposition or an underlying disease such growth hormone deficiency or inflammatory bowel disease.^7,8^ After undergoing evaluation for underlying pathology, a subset of children evaluated for short stature are found to have heights shorter than expected in the absence of an identified pathology or other explanation and are classified as having idiopathic short stature (ISS). This diagnosis can lead to extended periods of surveillance and testing and resultant parental and child anxieties about a potentially undiagnosed underlying disease.^9,10^ One possible explanation for an ISS diagnosis is a genetic predisposition to shorter stature that is not captured by traditional means. Currently, a child’s genetically predicted height is estimated based on their mother’s and father’s height. However, some children may receive a skewed distribution of height-associated genetic variants from their parents, which could lead to an inaccurate height prediction. Supporting this hypothesis, studies of children and adults in population-based cohorts have shown that the combination parental heights and polygenic scores for height improves the accuracy of height predictions, as compared to parental heights alone.^11,12^ Thus, some children who inherit a skewed distribution could be incorrectly classified as having ISS.

We hypothesized that a PGS_height_ could aid in identifying children with ISS who have an underestimated polygenic predisposition to shorter height and distinguish them from children with short stature due to an underlying disease. We first validated the association between the PGS_height_ and short stature using a pediatric cohort. We then tested whether children evaluated for unexplained short stature have a benign genetic predisposition to short stature and whether the PGS_height_ could improve discrimination between ISS and pathologic short stature when compared to mid-parental height (MPH).

## Methods

### Study populations

All data for participants were obtained from BioVU, a DNA-linked biobank linked to a deidentified copy of the electronic health record (EHR).^13^ BioVU comprises discarded blood samples from approximately 325,000 participants receiving healthcare at Vanderbilt University Medical Center (VUMC). For pediatric participants, parental permission is required prior to enrollment. These studies were evaluated by the VUMC Institutional Review Board (IRB) and determined to be non-human subjects research.

### Overall BioVU cohorts

These cohorts were curated to test the associations between the PGS_height_ and measured height in the BioVU population. 70,467 genotyped participants of European ancestry were identified. Participants with an ICD code for a potential pathologic cause of short stature were excluded. ICD codes for exclusion are listed in **Table S1**. For the adult cohort, participants were excluded if they had fewer than 2 height measurements between the ages of 20 and 70. For the pediatric cohort, participants were excluded if they had fewer than 2 height measurements between the ages of 2 and 19.

### Short stature cohort

The study cohort was derived from a set of 758 children who were evaluated by a pediatric endocrinologist on the same day that an ICD-9 or ICD-10 code for short stature appeared in their clinical record. ICD codes used to select this cohort are listed in **Table S2**. Participants were excluded prior to chart review if they had a known genetic syndrome, were born severely preterm (less than 32 weeks) or had prior treatment with growth hormone. After chart review, participants were excluded if the short stature diagnosis was inconclusive (diagnosis with 2 or more subtypes or lost to follow-up) or if they were missing initial visit or parental heights.

### Clinical data

For each participant, all endocrinology notes were manually reviewed by a medical student (JPS) and a clinician (JDM) in consultation with a pediatric endocrinologist (JHS). The final diagnosis assigned by the evaluating endocrinologist was extracted through review of clinical notes. Diagnostic groups analyzed in this study were short stature secondary to pathology (i.e., pathologic short stature), ISS, and the two benign causes of short stature: familial short stature and delayed puberty. Pathologic short stature included children with the following diagnoses: growth hormone deficiency, syndromic short stature, short stature secondary to systemic disease, undernutrition (including failure to thrive), small for gestational age, and chronic corticosteroid use. Children diagnosed with growth hormone deficiency but with normal growth hormone (GH) stimulation results (maximum GH response ≥ 10ng/ml) were re-categorized as ISS.

The “initial visit height” was the height measured on the same day as the initial endocrinology visit. If not available on the same day, the height value occurring within one year and closest to the initial visit date was selected. The R package childsds was used to convert initial visit height measurements to age- and sex-specific standard deviation scores (SDS) using the Centers for Disease Control and Prevention’s (CDC) growth reference standards.^14^

Parental heights were extracted through chart reviews. These heights were converted to sex-specific SDS using the National Health and Nutrition Examination Survey (NHANES) cohorts as the reference standard for the adult population in the United States.^15^ For the SDS, 0 was defined as the mean height among NHANES adults of each participant’s sex and the standard deviation was defined as the standard deviation of the distribution in these adults. The NHANES data is further described in the **Supplemental Methods.** Mid-parental height (MPH) for a child was calculated as 0.72 × (average of parental height SDS), as recommended in guidelines from the Pediatric Endocrine Society.^16,17^

### Genetic data

Single-nucleotide polymorphism (SNP)-based genotyping was performed using the Illumina MEGA-Ex platform. SNPs were imputed to the Haplotype Reference Consortium (HRC) release 1.1 haplotypes using the Michigan Imputation Server.^18^ Closely related individuals were excluded by randomly removing one of each pair of individuals with pi-hat genetic relatedness greater than 0.2. Participants with genotype missingness greater than 2% were excluded as were participants with outlying heterozygosity defined as a heterozygosity more than 4 standard deviations from the mean. Genetic ancestry was determined by principal components (PCs) analysis in conjunction with HapMap reference populations.^19^ Within-ancestry PCs to adjust for residual population stratification were calculated using the SNPRelate R package.^20^

### Development of the PGS

These analyses utilized a previously validated PGS for adult height (PGS_height_) which was derived from a GWAS of over five million adults (76.8% European) across 281 studies.^12^ In that study, SNP effect estimates for the PGS study were generated using SBayesC.^21^ Weights specific to the European ancestry subset were downloaded from https://www.joelhirschhornlab.org/giant-consortium-results. Of the 1,098,854 SNPs with available weights, 1,096,598 (99.8%) were available in our imputed dataset (imputation R^2^ ≥ 0.7). The PGS_height_ was computed for each child by summing the product of each allele weight and the allele dosage across all SNPs. The PGS_height_ was then converted to SDS, defining 0 as the mean PGS_height_ SDS among the cohort of BioVU adults without an ICD code for a potentially pathologic cause of short stature as described earlier. The standard deviation for the SDS was defined as the standard deviation of the distribution in these adults. The pediatric population was not used to avoid selection bias due to an enrichment of patients undergoing evaluation for short stature in our biobank.

### Statistical analyses

Baseline characteristics of the cohorts were summarized as counts (frequencies) for binary variables and median (interquartile ranges [IQR]) for continuous variables.

In the adult BioVU cohort, multivariable linear regression models were used to test the association between a person’s PGS_height_ and the median value of all available height measurements for an individual, adjusting for the top 10 PCs. In the pediatric cohort, multivariable logistic regression models, also adjusting for the top 10 PCs, were used to test the association between the PGS_height_ and meeting the threshold for short stature, defined as a height ≥ 2 standard deviations below the mean.

In the short stature cohort, one-sample Wilcoxon rank sum tests were used to determine whether the median MPH and PGS_height_ were shorter than expected based on adult population averages (i.e., whether either SDS differed from 0). Tukey’s honest significant difference (HSD) test was used to estimate and test the significance of pairwise differences in the PGS_height_ SDS between short stature subtypes. To enable direct comparisons of predicted height estimates based on the PGS_height_ and MPH, the PGS_height_ SDS was converted to a predicted adult height using a model developed in the adult BioVU cohort (see the **Supplemental Methods** for full details). Paired-sample Wilcoxon rank-sum tests were utilized to test the within-person difference between the MPH-based and PGS_height_-based adult height predictions by diagnostic sub-group. Additional sub-group analyses were performed among the participants diagnosed with pathologic short stature, stratifying by the underlying disease process driving short stature.

After restricting to the subset of participants diagnosed with either ISS or pathologic short stature, we tested whether the PGS_height_ could improve discrimination between children with ISS and pathologic short stature. To test the association of the PGS_height_ with receiving an ISS diagnosis, two multivariable logistic regression models were fit: 1) a baseline model that included a child’s sex, age at the initial visit, and the top 10 within-ancestry PCs and 2) the baseline model with the addition of the PGS_height_. This PGS_height_ model was used to generate predicted probabilities of ISS (versus pathologic short stature) for each participant. AUCs were then derived from these models and their 95% confidence intervals were computed by bootstrapping with 5,000 replicates. The improvement in discrimination with addition of the PGS_height_ was assessed by calculating the change in AUC (reported as Δ[AUC]), computing 95% confidence intervals by bootstrapping with 5,000 replicates. In a sensitivity analysis, the baseline model was additionally adjusted for initial visit height. Additional sensitivity analyses examined subgroups of children: 1) falling outside of MPH-based genetic height predictions (discrepancy between child’s measured height SDS and MPH SDS ≥ 2 s.d.); and 2) meeting the clinical definition of short stature at the initial visit (height SDS ≤ −2).^17^ These analyses utilized the R package pROC for ROC curve analysis and the packages boot and boot.pval for bootstrapping.

In association analyses, statistical tests were two-sided and a p-value < 0.05 was considered significant. In discrimination analyses, a change in c-statistic with a bootstrapped p-value < 0.05 was considered significant. All analyses were performed using R version 4.0.2.

## Results

### A lower PGS_height_ is associated with a higher likelihood of pediatric short stature

**Figure 1:**
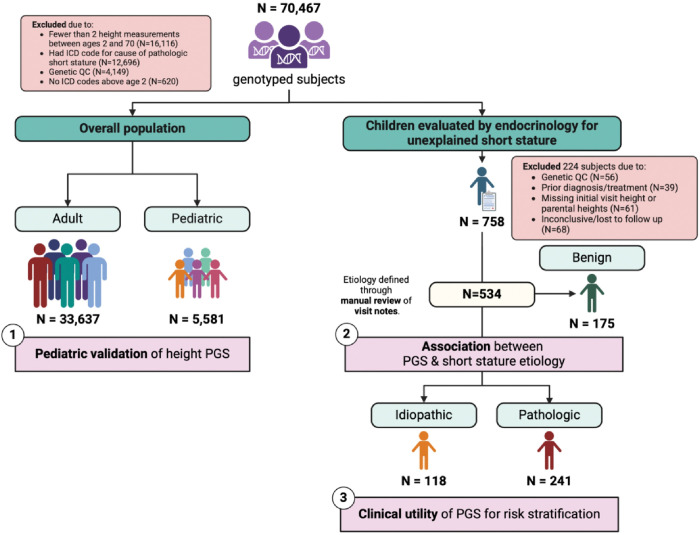
Outline of study populations and aims. ‘Benign’ refers to the causes of short stature that are not attributable to underlying disease: familial short stature or short stature due to delayed puberty.

**Figure 2:**
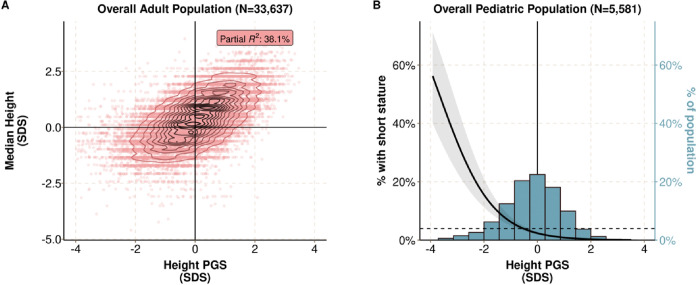
A polygenic predisposition to height (PGS_height_) is associated with adult height and prevalent short stature in BioVU. **A)** Scatter plot comparing the PGS_height_ to age- and sex-normalized heights among adults (n=33,637). Height was defined as the median of all of a given subject’s height measurements. The partial R2 is from a linear model adjusting for the top 10 within-ancestry principal components. **B)** Histogram of the PGS_height_ distribution in the pediatric population and the association between the PGS_height_ and clinical short stature (median height 2 or more standard deviations below the reference standard) (n=221 [4.0% of 5,581]). The dashed line reflects the prevalence of short stature in the population and the left y-axis reflects the predicted probability of short stature with a lower PGS_height_. The right y-axis reflects the percent of the pediatric population with a given PGS_height_.

To validate the PGS_height_, we identified a cohort of 33,637 adult and 5,581 pediatric genotyped participants in BioVU who did not have a diagnosis of a disease that can lead to short stature (Fig. 1, **Table S3**). The majority of participants were female (60.0% in the adult population, 57.1% in the pediatric population). The mean age was 49.6 years (SD 15.0) for adults and 13.3 years (6.3) for children. Among the adult population, there was a median of 10 (interquartile range [IQR]: 5 to 22) height measurements per person over 11.3 (5 to 17.2) years. Among the pediatric population, there was a median of 7 (IQR: 3 to 16) height measurements per person over 10.2 (5.5 to 15.5) years. The PGS_height_ explained 38.1% of height variation among adults (Fig. 2A). Among children, the PGS_height_ was inversely associated with meeting the criteria for short stature, defined as a median height more than 2 standard deviations below the expected mean for the patient’s age and sex (odds ratio (OR): 0.36 (0.31–0.43); p = 2×10^−35^) (Fig. 2B).

In sum, the PGS_height_ explained a significant portion of height variance in adults, and a polygenic predisposition to shorter height was associated with an increased likelihood of meeting the diagnostic criteria for short stature among children.

### Children with familial short stature and ISS have a lower PGS_height_ than other sub-groups

There were 534 children who underwent an evaluation in pediatric endocrinology clinic for unexplained short stature (Fig. 1). The median follow-up time was 3 (IQR, 2 to 9) visits over a median 1.9 (0.3 to 4.7) years. Within this cohort, 241 (45.1%) children were diagnosed with short stature secondary to underlying disease, 118 (22.1%) with ISS and 175 (32.8%) with the benign subtypes: delayed puberty (N = 140, 26.2%) and familial short stature (N = 35, 6.6%). Of the children with pathologic short stature, the most common diagnoses were growth hormone deficiency (N = 94, 39.0%), short stature secondary to systemic disease (N = 75, 31.1%), and syndromic short stature (N = 35, 14.5%) (Table 1). Children with short stature secondary to systemic disease were most commonly diagnosed with inflammatory bowel disease (N = 23 of 75, 30.7%), cystic fibrosis (N = 7, 9.3%), and celiac disease (N = 7, 9.3%). The most common causes of syndromic short stature were neurofibromatosis type 1 (N = 4 of 35, 11.4%) and mosaic Turner syndrome (N = 3, 8.6%).

**Figure 3:**
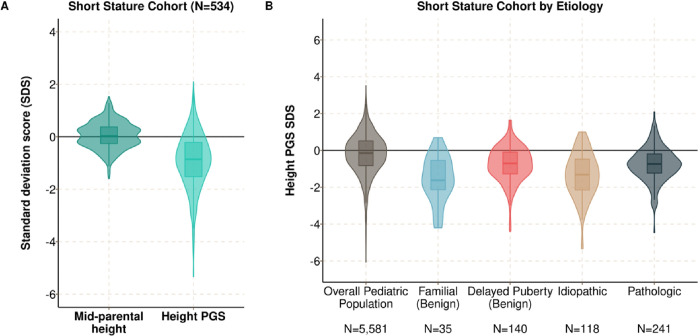
Children referred for short stature have a polygenic predisposition to shorter height, which is greatest for familial short stature and ISS. **A)** Violin plots showing the PGS_height_ and the MPH SDS among participants in the short stature. The MPH SDS reflects the sex-adjusted standardized height relative to the NHANES reference standard, while the PGS_height_ SDS reflects the standardized PGS_height_ relative to the BioVU adult population. **B)** Violin plots comparing PGS_height_ SDS by short stature etiology in the short stature cohort, The overall pediatric population is included as a reference.

The median height SDS at the initial visit height was − 2.05 (IQR, −2.46 to −1.59) and the median PGS_height_ SDS was − 0.87 (IQR, −1.54 to −0.23) (Fig. 3A). We then compared the initial visit height and the PGS_height_ SDS between diagnostic subtypes using Tukey’s HSD. We first tested whether the height and PGS_height_ differed between children diagnosed with ISS and familial short stature. Children diagnosed with ISS were shorter on average than children with familial short stature (ΔPGS_height_ = −0.48 s.d. [−0.75 to −0.22], Tukey’s *p* = < 0.001) but the PGS_height_ did not differ (0.19 [−0.31 to 0.70], *p* = 0.75) (Fig. 3B and S1). We then tested whether the height or the PGS_height_ was different between children with ISS and pathologic short stature. Height did not differ between children with ISS and pathologic short stature (0.07 [−0.22 to 0.09], *p* = 0.68), but children with ISS had a lower PGS_height_ than children with pathologic short stature (−0.60 [−0.89 to −0.31], *p* < 0.001).

These results demonstrate that children diagnosed with ISS and pathologic short stature have similar initial visit heights, but those with ISS have a larger polygenic predisposition to shorter heights. In contrast, the children with ISS and familial short stature had similar predispositions.

### Adult height predictions based on MPH and PGS_height_ are most discordant in ISS

**Figure 4:**
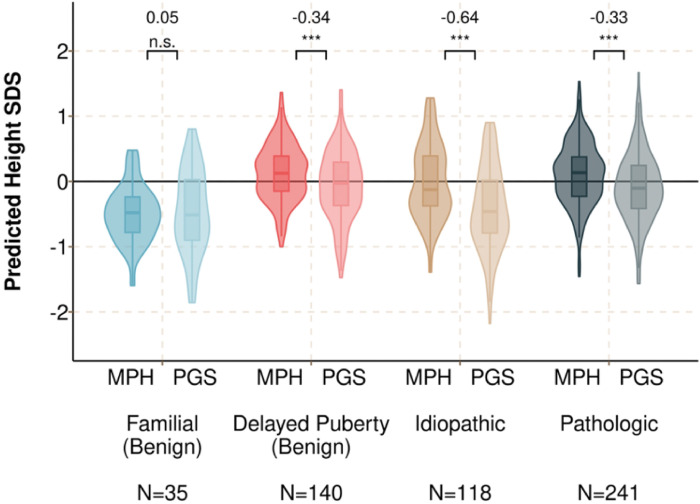
Predicted heights based on the PGS_height_ are lower, compared to MPH-based heights, except for children with familial short stature. Violin plots comparing height predictions by method (MPH and PGS_height_) for each sub-group based on short stature etiology. The PGS_height_ was converted to a predicted adult height by modelling height as a function of PGS_height_ and 10 principal components in BioVU adults. Cohen’s d statistics (shown above each pair) were used to estimate the standardized mean difference in height predictions generated using the PGS_height_ and MPH. Paired samples Wilcoxon rank sum tests were used to test the significance of within-person differences. ***: Wilcoxon p<0.001. n.s.: p>0.05.

We then tested whether genetic height estimated using parental heights (i.e., MPH) were discordant from the PGS_height_. Discordance between the measures could indicate that the MPH is misestimating height, which could lead to misclassification as having ISS. To compare adult height predictions based on the PGS_height_ and MPH on the same unit scale, the PGS_height_ SDS was converted to a predicted adult height SDS for each child (see **Supplemental Material**). Among children diagnosed with familial short stature, PGS-predicted adult height and MPH-predicted adult height were largely concordant (Cohen’s *d* for standardized mean difference = 0.05; *p* = 0.46) (Fig. 4). However, predicted heights based on the PGS_height_ were lower, compared to MPH, among children with ISS (*d* = −0.64; *p* = 4.0×10^−18^) and pathologic short stature (*d* = −0.33; *p* = 1.3×10^−13^). Within the pathologic group, height predictions were lower when using PGS_height_ for children diagnosed with growth hormone deficiency (N = 94) and systemic disease (N = 75) but not in the genetic syndrome sub-group (N = 35) (**Figure S2**).

In summary, predicted heights based on MPH and PGS_height_ are most discordant among children with ISS, suggesting that MPH is underestimating their polygenic predisposition to lower height.

### PGS_height_ improves model discrimination between ISS and pathologic short stature

**Figure 5:**
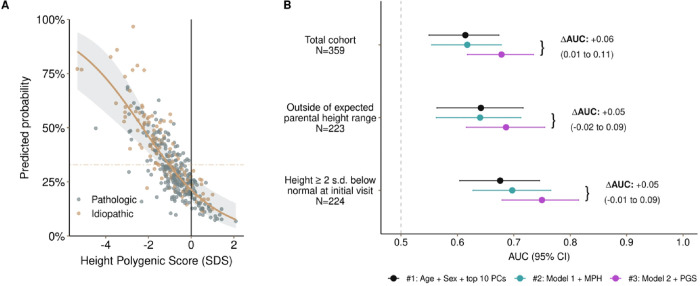
The PGSheight improves model discrimination between children with ISS versus pathologic short stature. **A)** Scatter plot showing the relationship between the PGS_height_ and receiving a diagnosis of ISS in the ISS/pathologic-only cohort. Logistic regression, adjusting for 10 PCs, was used to generate predicted probabilities of ISS The gold line denotes the baseline probability of being classified as having ISS. **B)** Forest plot comparing discrimination (AUC) between ISS and pathologic short stature when using a model with only age, sex, and 10 PCs (black), with addition of the MPH (blue) and with addition of the PGS_height_ (purple). Additional sensitivity analyses examined subgroups of children: 1) outside of the expected parental height range (discrepancy between child’s measured and MPH ≥ 2 standard deviations) and 2) meeting the clinical definition of short stature at the initial visit (height SDS ≤ −2).

We then examined whether the PGS_height_ would discriminate between children with ISS and pathologic short stature. We utilized multivariable logistic regression to test the association between PGS_height_ and a diagnosis of ISS versus pathologic short stature (N = 241 [67%] pathologic, 118 [33%] ISS), adjusting for age, sex, and local ancestry. The PGS_height_ was inversely associated with the likelihood of ISS diagnosis (odds ratio [OR], 0.56 [0.04–0.73]; *P* = 3.0×10^−5^) (Fig. 5A). This finding indicates that a polygenic predisposition to shorter height was associated with a lower likelihood of identifying underlying pathology. To examine improvement in discrimination, we tested the addition of the PGS_height_ to a baseline model that included MPH. Adding the PGS_height_ improved the c-statistic from 0.61 to 0.68 (ΔAUC = + 0.06 [95% CI, 0.01–0.10], *P* = 0.03) (Fig. 5B, Table S4). In sensitivity analyses, the ΔAUC remained significant when additionally adjusting for a child’s initial visit height (+ 0.06 [0.01–0.11]; *P* = 0.02) (**Table S5**). In sub-group analyses, similar improvements in the c-statistics were seen in analyses of children with height ≥ 2 standard deviations below the population mean (N = 224; +0.05 [−0.01, 0.09]) and children with an initial height ≥ 2 standard deviations below the expected MPH (N = 223; +0.05 [−0.02, 0.09]) (**Table S4 and S5; Figure S3**).

In sum, the PGS_height_ improved discrimination for a predictive model of ISS versus pathologic short stature beyond MPH.

## Discussion

We examined the utility of incorporating a PGS_height_ in the evaluation of pediatric patients with short stature. Children with ISS had a comparable PGS-predicted height as children with familial (genetic) short stature. The primary diagnostic difference between these groups is that children with familial short stature manifest heights in line with those of their parents, while children with ISS do not. To further explore this difference, we directly compared height predictions based on the PGS_height_ to those based on mid-parental height. Using mid-parental height, children with ISS were predicted to have heights near the population mean, but using the PGS_height_, children were predicted to be significantly below the mean. To test the clinical implications of this discordance, we examined whether the PGS_height_ could distinguish between children diagnosed with ISS and pathologic short stature. The PGS_height_ was able to modestly discriminate the two groups, while mid-parental height could not. These findings suggest that mid-parental height estimates may miss genetic factors captured by the PGS_height_, thereby leading to a misclassified ISS diagnosis. Incorporating polygenic height predictors could improve diagnostic accuracy, ultimately improving clinical outcomes for children with ISS.

Studies in prospective, population-based cohorts have shown that polygenic scores for height can improve adult height predictions and identify children at risk for short stature in adulthood.^11,12^ Our study extends this work to children with short stature who are undergoing a diagnostic evaluation seeking an explanation for their height. Short stature comprises a heterogeneous collection of diagnoses, ranging from benign conditions such as familial short stature to underlying diseases. Consequently, diagnostic evaluations can be extensive, but leave many children without an explanation.^9,22,23^

An adult height estimate based on parental heights (MPH) is typically used to estimate a child’s genetically determined adult height potential. This study highlights the potential limitations of MPH and suggests that it may not accurately measure polygenic drivers of growth for all children presenting for evaluation. This discordance may be particularly apparent among children referred for an endocrine evaluation as one indication for referral is discordance between the child’s height and their MPH. Consistently, children with ISS in this cohort had average parental heights, so short stature was not expected by the clinician based on mid-parental height alone.

The inherent uncertainty of an ISS diagnosis can often lead to prolonged monitoring for latent disease that has not revealed itself.^24^ In this study, we show that the PGS_height_, but not the MPH, improved discrimination of children with underlying disease from those children that remain with ISS even after a specialist’s evaluation. While significant, the magnitude of the improvements were relatively modest. The most likely explanation for the modest improvement is that ISS represents a heterogeneous collection of etiologies including rare genetic variation or sub-clinical disease.^25^ An unmeasured polygenic predisposition could be considered an additional, testable cause of short stature in this population. A polygenic approach to height estimation could provide some children with ISS an explanation for their short stature, allowing for appropriate de-escalation of care for disease monitoring.

Discordance between parental and polygenic height prediction could have many explanations including misestimation of parental heights due to inaccurate reporting or misattributed paternity.^26^ In one study, inaccurate self-reporting of parental heights was common, with 30% of couples having a self-reported MPH that was more than 2 centimeters different than measured MPH.^27,28^ In these instances, the PGS_height_ could be a valuable addition to a diagnostic algorithm, in particular for those children diagnosed with ISS. Another application would be for adopted children with unknown parental heights, for whom MPH estimates are not available.

Recessive genetic variation or de novo genetic mutations are well recognized but often underdiagnosed causes of ISS. A recent meta-analysis has shown that exome sequencing and chromosomal microarray can identify a genetic etiology in 27.1% and 13.6% of patients, respectively, that were previously diagnosed with ISS.^25^ In children with ISS, the PGS_height_ in conjunction with MPH could identify children in whom additive genetic variation is unlikely to be an explanation and who are more likely to carry rare and potentially clinically actionable variants. This approach could improve the yield of clinical sequencing studies for children with ISS.^29^

This study’s strengths include the use of expert-adjudicated real-world diagnosis labels and a polygenic score derived from the largest genome-wide association study to date. There are also limitations. These data are from a tertiary medical center and represent a highly selected population that may differ from typical pediatric populations. Additionally, the PGS_height_ was derived from a European ancestry population, limiting its applicability across different genetic ancestries.^12^ We were unable to assess associations among children of non-European ancestries as there were fewer than 10 total cases of ISS in children of African, Asian, and Hispanic ancestries. Future research should aim to validate these findings in more diverse populations to enhance the generalizability of PGS_height_ across different genetic ancestries. Additionally, prospective studies could assess the real-world impact of incorporating PGS_height_ into diagnostic algorithms for short stature.

In summary, we show that the PGS_height_ measures a polygenic predisposition to shorter height not fully captured by MPH and can modestly discriminate children with ISS from those with short stature due to pathology. Incorporating the PGS_height_ into clinical practice not only could provide reassurance to children, parents, and clinicians by helping to identify the etiology of ISS, but also facilitates more targeted and efficient care, potentially allowing for the de-escalation of unnecessary diagnostic procedures.

## Figures and Tables

**Table 1:** Descriptive statistics for short stature cohort.

Characteristic	Outcome
**N**	534
**Female, No. (%)**	28.3%
**Age (years) at initial visit**	
Median (IQR)	10.7 (7.5 to 13.2)
**Height SDS at initial visit^a^**	
Median (IQR)	−2.05 (−2.46 to −1.59)
Height SDS ≤ −2, No. (%)	289 (54.1%)
Deviation from MPH ≥ 2 s.d., No. (%)	285 (53.4%)
**Genetic height predictors, median (IQR)**	
Mid-parental height SDS^b^	0.03 (−0.26 to 0.38)
PGS_height_ SDS^c^ (z)	−0.86 (−1.52 to −0.22)
**Length of follow-up**	
Number of visits, median (IQR)	3 (2 to 9)
Number of years, median (IQR)	1.9 (0.3 to 4.7)
**Testing, No. (%)**	
Bone age	494 (92.5%)
IGF-1	484 (90.6%)
Growth hormone stimulation	187 (35.0%)
**Initiated growth hormone treatment, No. (%)**	215 (40.3%)
**Etiology of short stature**	
Familial	35 (6.6%)
Delayed puberty	140 (26.2%)
Idiopathic (ISS)	118 (22.1%)
Pathologic	241 (45.1%)
Growth hormone deficiency	94 (17.6%)
Systemic disease	75 (14.0%)
Syndromic	35 (6.6%)
Undernutrition	19 (3.6%)
Small for gestational age	13 (2.4%)
Corticosteroid use	5 (0.9%)

a. Height SDS was calculated by standardizing pediatric heights to CDC reference standards.

b. Mid-parental height was standardized to the NHANES adult population as described in the Supplemental Material.

c. PGS_height_ SDS was calculated by standardizing to the adult BioVU dataset, specifying a mean of 0 and a standard deviation of 1.

## Data Availability

Subject-level access to BioVU clinical and genetic data is controlled by the BioVU data repository (https://victr.vumc.org/biovu-description/). Upon publication, data sets of subject-level phenotype data and corresponding data dictionaries to replicate the primary findings presented here for research purposes will be made available upon request from the repository (biovu@vumc.org). Vetting for use of BioVU data includes institutional IRB approval, data use agreements, and administrative and scientific reviews.
